# Supplementary figures and images for: Trends in COVID-19 case-fatality rates in Brazilian public hospitals: A longitudinal cohort of 398,063 hospital admissions from 1st March to 3rd October 2020
Source: PLoS One. 2021 Jul 16;16(7):e0254633. doi: 10.1371/journal.pone.0254633 (PMC8284655; doi:10.1371/journal.pone.0254633)

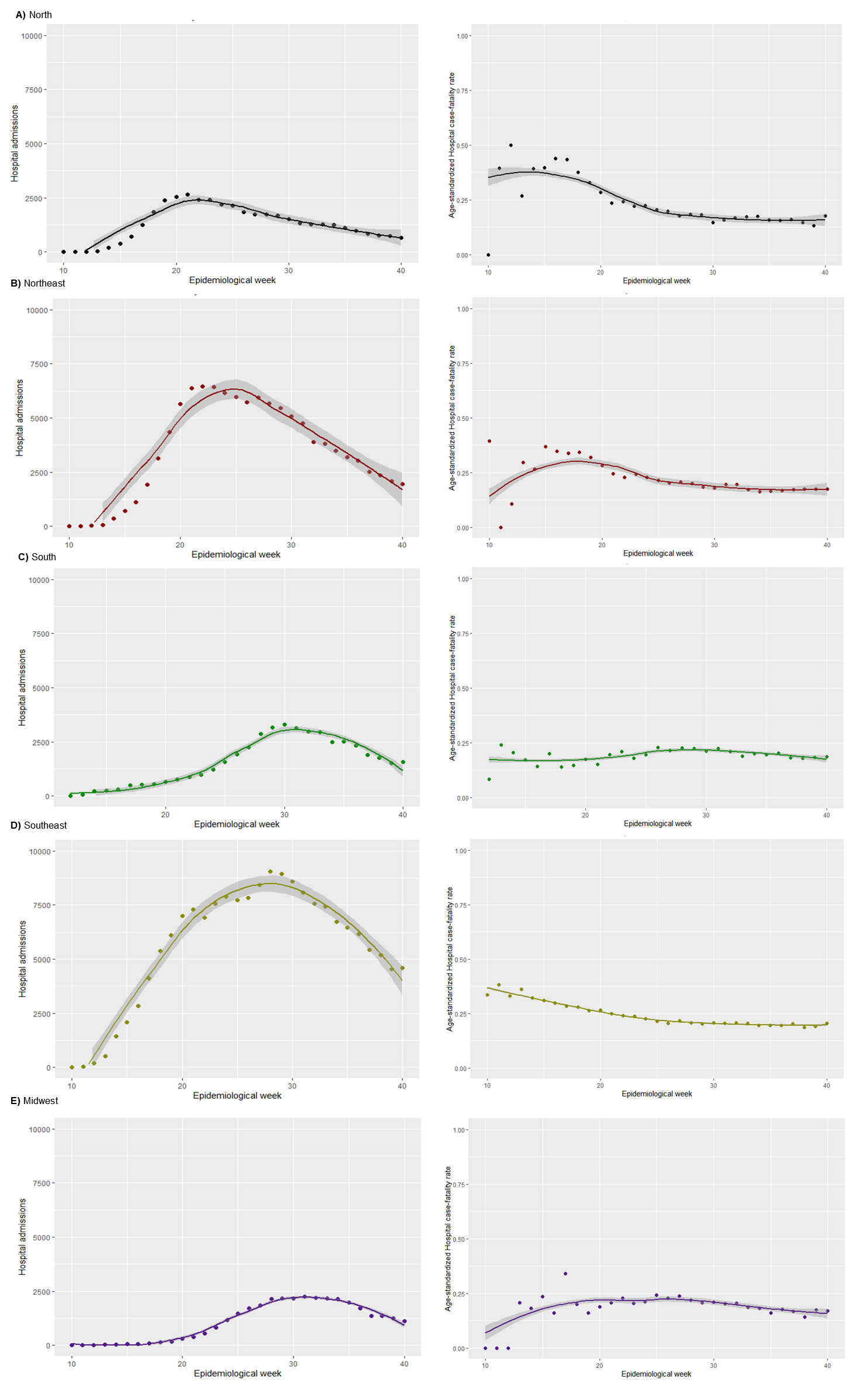

Supplement: S1 Fig — Timeline of 398,063 COVID-19-related hospital admissions (left) and age-standardized hospital case-fatality rates (right) stratified by geographic region during epidemiological weeks 10 to 40, Brazil, 2020. (TIF) [file pone.0254633.s001.tif]

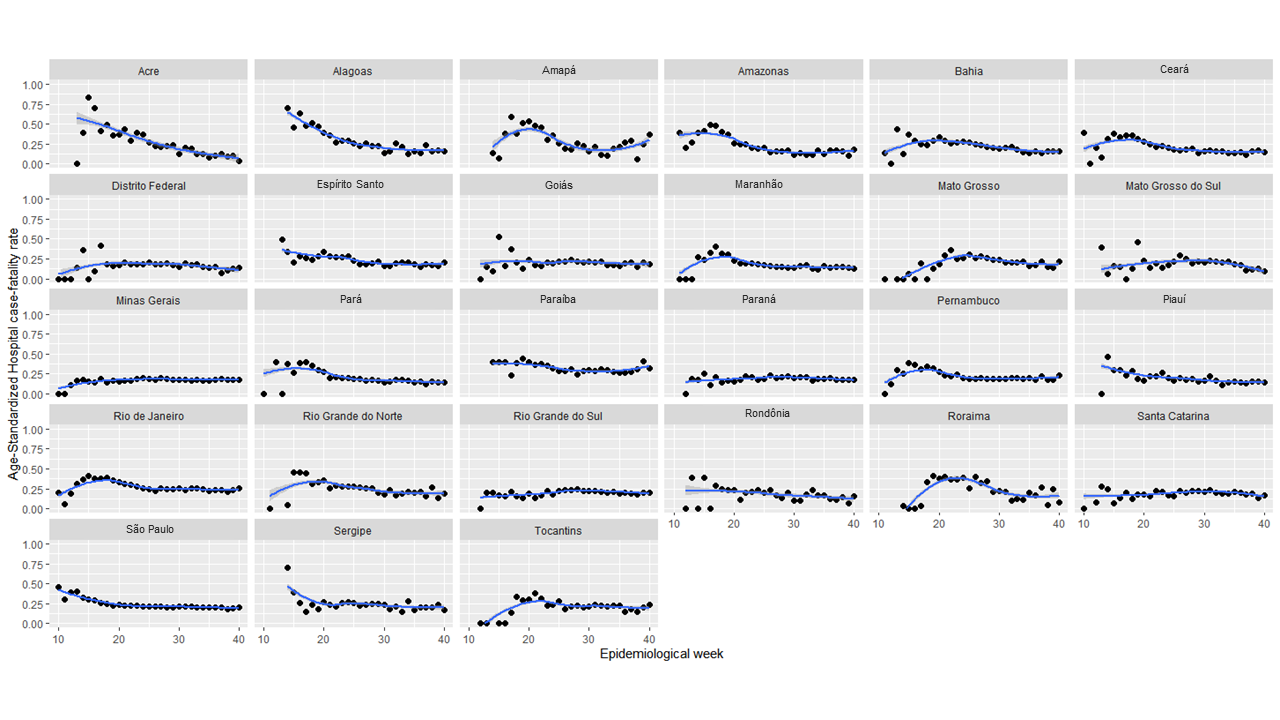

Supplement: S2 Fig — (TIF) [file pone.0254633.s002.tif]

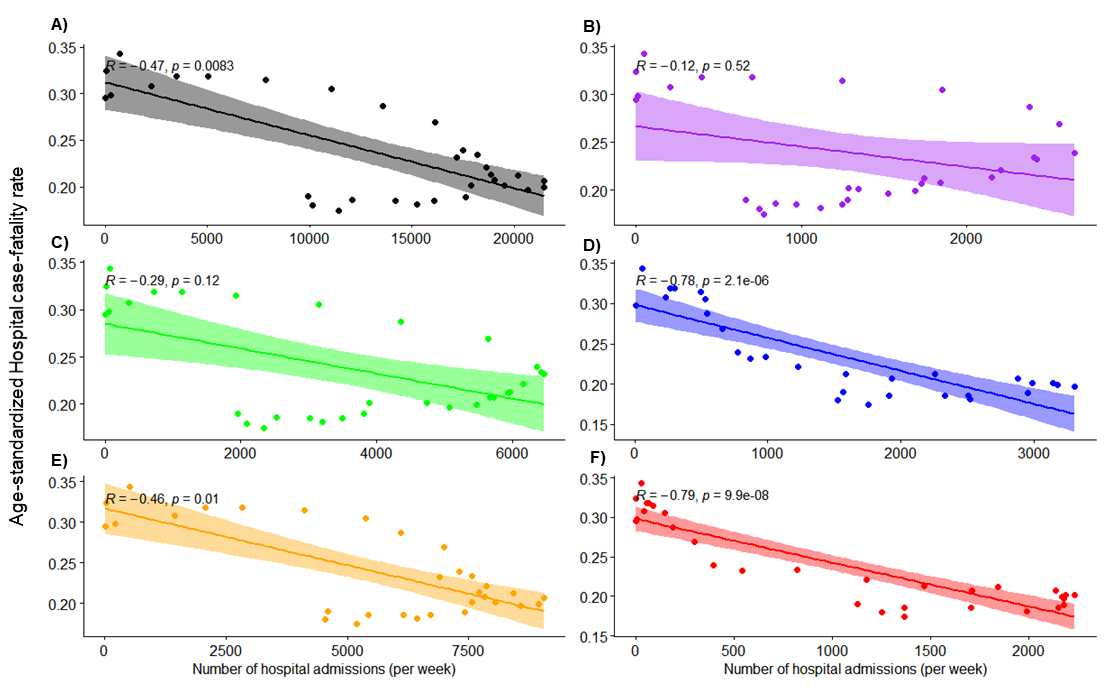

Supplement: S3 Fig — Correlation analysis between age-standardized hospital case-fatality rates and the number of hospital admissions per week stratified by A) All regions, B) North, C) Northeast, D) South, E) Southeast and F) Midwest region during epidemiological weeks 10 to 40, Brazil, 2020. (TIF) [file pone.0254633.s003.tif]
