# Supplementary material for: Trends in COVID-19 case-fatality rates in Brazilian public hospitals: A longitudinal cohort of 398,063 hospital admissions from 1st March to 3rd October 2020
Source: PLoS One. 2021 Jul 16;16(7):e0254633. doi: 10.1371/journal.pone.0254633 (PMC8284655; doi:10.1371/journal.pone.0254633)
Supplement: S1 File — (DOCX) [file pone.0254633.s004.docx]

**S1 File**


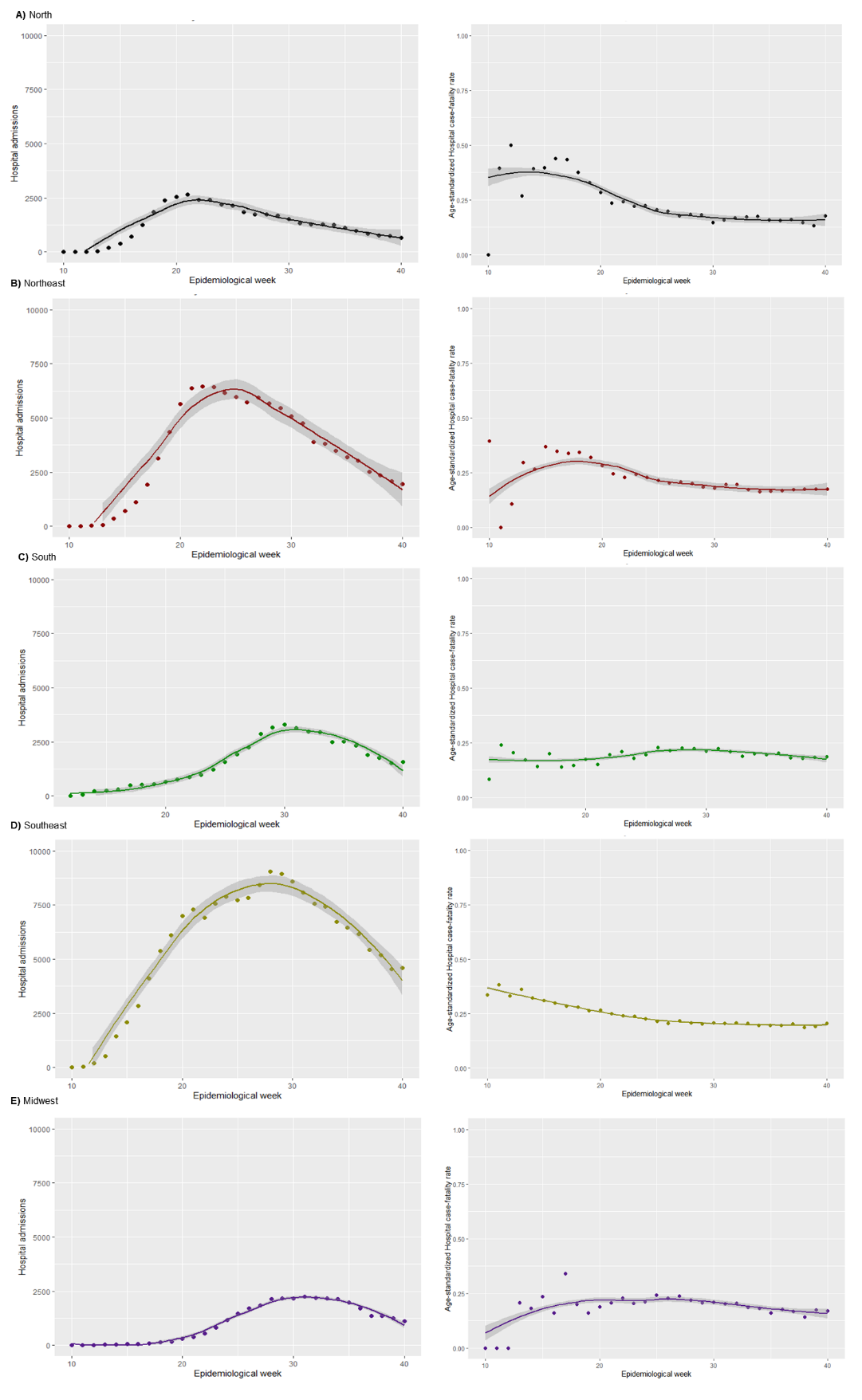


**S1 Fig** – Timeline of 398,063 COVID-19-related hospital admissions (left) and age-standardized hospital case-fatality rates (right) stratified by geographic region during epidemiological weeks 10 to 40, Brazil, 2020.


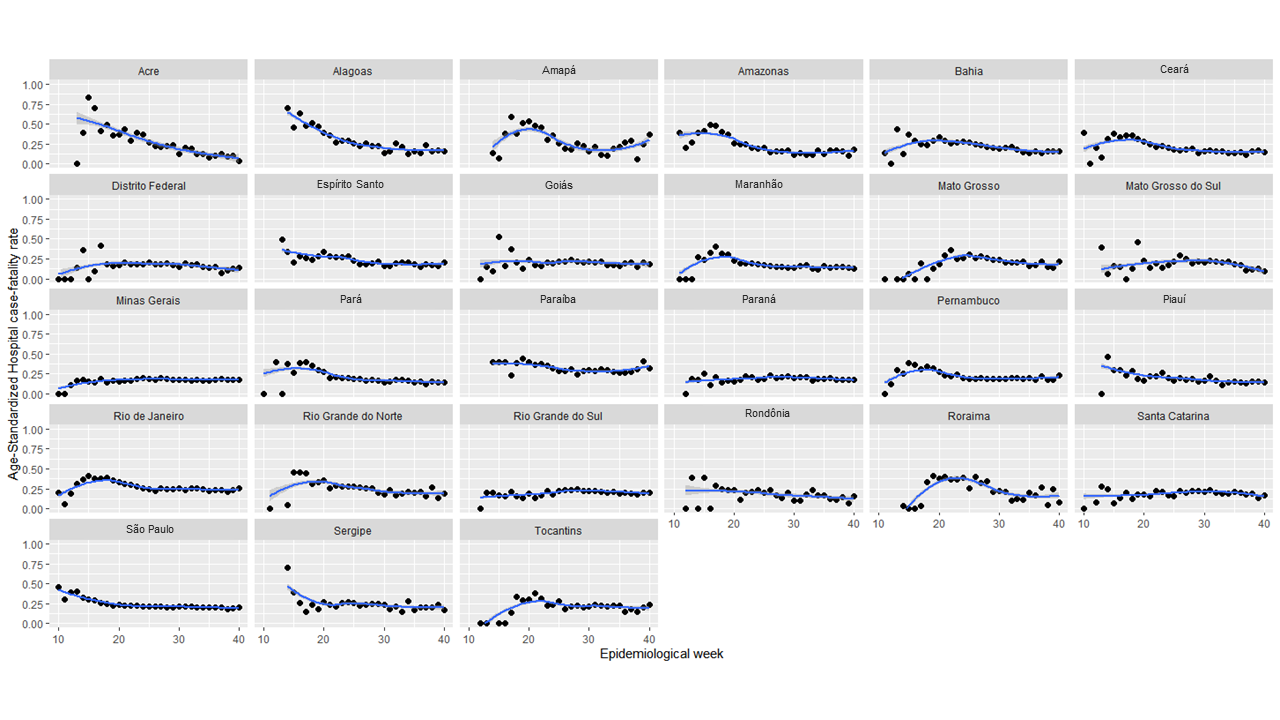


**S2 Fig** – Age-standardized hospital case-fatality rates stratified by Brazilian states during epidemiological weeks 10 to 40, Brazil, 2020


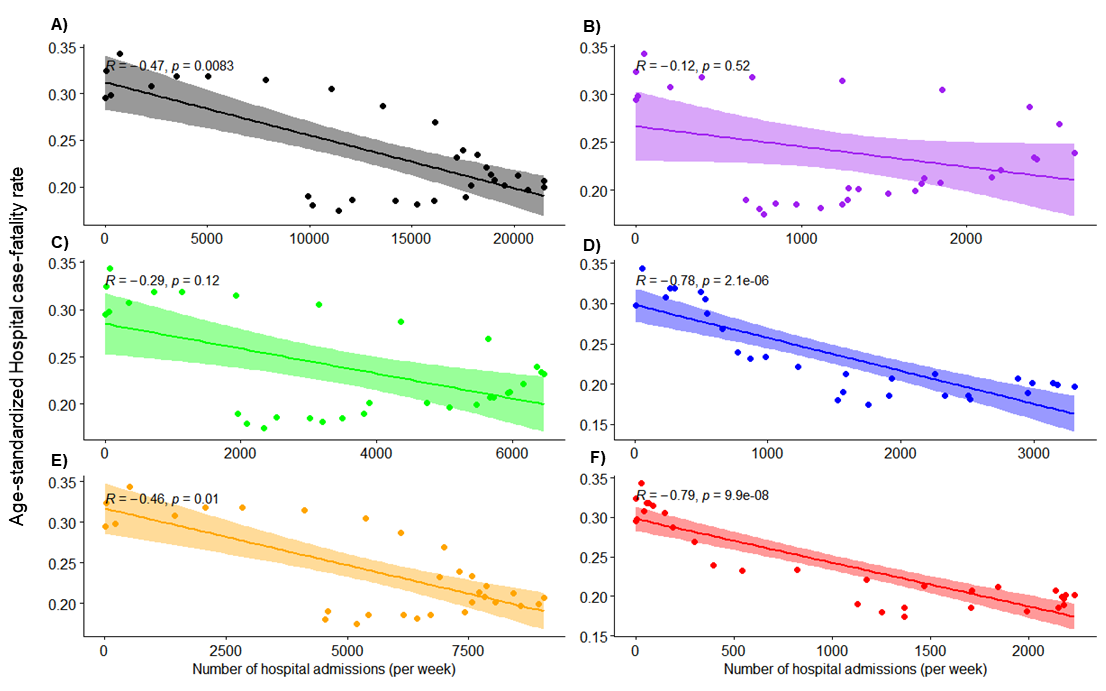


**S3 Fig** – Correlation analysis between age-standardized hospital case-fatality rates and the number of hospital admissions per week stratified by A) All regions, B) North, C) Northeast, D) South, E) Southeast and F) Midwest region during epidemiological weeks 10 to 40, Brazil, 2020

**S1 Table**: Variable Description

| **Variable/Category** | **Variable description** |
| --- | --- |
| Ethnicity /White | Self-declared as white |
| Ethnicity /Multiracial | Self-declared as multiracial |
| Ethnicity /Black | Self-declared as black |
| Ethnicity /Asian | Self-declared as Asian |
| Ethnicity / Native Brazilians (indigenous) | Self-declared as indigenous |
| Ethnicity /Not declared | Did not declare race |
| Female patient | Female patient |
| Male patient | Male patient |
| COVID-19 as the main disease | Main diagnosis was COVID-19 |
| High complexity case | Admission treated with procedures that, in the context SUS, involve high technology and high cost |
| ICU utilization | Used ICU bed |
| ≤< 19 years | Up to 19 years old |
| 20 - 39 year | Between 20 and 39 years old |
| 40 - 59 years | Between 40 and 59 years old |
| 60 - 79 years | Between 60 and 79 years old |
| 80 + years | 80 years or older |
| Admission in March | Hospitalized in March |
| Admission in April | Hospitalized in April |
| Admission in May | Hospitalized in May |
| Admission in June | Hospitalized in June |
| Admission in July | Hospitalized in July |
| Admission in August | Hospitalized in August |
| Admission in September | Hospitalized in September |
| Admission in October | Hospitalized in October |
| Comorbidity Obesity | ICD-10 code: E66 |
| Comorbidity Bacterial infection | ICD-10 codes: A27-A49 |
| Comorbidity Cancer | ICD-10 codes: C01-C49 and D00-D48 |
| Comorbidity Diabetes | ICD-10 codes: E10-E14 |
| Comorbidity Cardiac disease | ICD-10 codes: I10-I99 |
| Comorbidity Renal insufficiency | ICD-10 code: N17 |
| Comorbidity Respiratory disease | ICD-10 codes: J44-J45 |
| Comorbidity Symptoms, signs and abnormal clinical and laboratory findings, not elsewhere classified | ICD-10 codes: R00-R99 |
| Comorbidity HIV | ICD-10 codes:B20-B24 |

**S2 Table.** Crude hospital case fatality rate of 398,063 COVID-19-related hospital admissions stratified by sex, age, comorbidities, ethnicity, length of stay, ICU need and geographic region during epidemiological weeks 10 to 40, Brazil, 2020.

| week | All | Sex | | Age range | | | | | Ethnicity | | | | | Length of stay | | | ICU admission | | Region | | | | |
| --- | --- | --- | --- | --- | --- | --- | --- | --- | --- | --- | --- | --- | --- | --- | --- | --- | --- | --- | --- | --- | --- | --- | --- |
|  |  | Female | Male | 00 - 19 | 20 - 39 | 40 - 59 | 60 - 79 | 80+ | Black | Brown | Indigenous | White | Yellow | 7 or less | Between 7 and 14 | More than 14 | No | Yes | Midwest | North | Northeast | South | Southeast |
| 10 | 37.5% | 40.0% | 36.4% | 50.0% | NA | 16.7% | 57.1% | NA | NA | 57.1% | NA | 25.0% | NA | NA | NA | 50.0% | 22.2% | 57.1% | NA | NA | 50.0% | NA | 41.7% |
| 11 | 22.5% | 15.4% | 35.7% | NA | 22.2% | 12.5% | 31.3% | 100.0% | 100.0% | 21.1% | NA | 26.7% | NA | 13.0% | 50.0% | 33.3% | 18.8% | 37.5% | NA | 100.0% | NA | NA | 29.6% |
| 12 | 24.6% | 23.8% | 25.4% | 5.4% | 10.0% | 12.3% | 42.7% | 57.1% | 30.4% | 24.5% | NA | 27.6% | 20.0% | 18.9% | 34.8% | 33.9% | 13.9% | 47.3% | NA | 27.3% | 8.2% | 15.4% | 29.3% |
| 13 | 29.4% | 28.1% | 30.4% | 2.7% | 11.7% | 30.2% | 42.2% | 50.0% | 31.1% | 29.4% | NA | 29.0% | 9.1% | 20.2% | 44.4% | 42.9% | 19.7% | 50.4% | 18.5% | 27.7% | 20.9% | 19.6% | 32.2% |
| 14 | 28.5% | 25.9% | 30.3% | 10.0% | 12.3% | 20.1% | 40.7% | 49.1% | 36.0% | 30.0% | 50.0% | 27.8% | 12.5% | 25.3% | 29.5% | 35.3% | 19.5% | 46.9% | 13.2% | 34.1% | 23.5% | 18.2% | 31.0% |
| 15 | 30.2% | 28.4% | 31.6% | 9.5% | 12.9% | 22.5% | 40.1% | 52.6% | 43.6% | 31.7% | 20.0% | 25.3% | 23.9% | 26.2% | 32.5% | 38.6% | 20.3% | 52.5% | 21.4% | 36.1% | 34.4% | 15.9% | 29.8% |
| 16 | 30.2% | 27.6% | 32.2% | 7.7% | 11.9% | 22.3% | 40.8% | 52.5% | 34.9% | 32.4% | 50.0% | 26.3% | 24.7% | 27.2% | 30.9% | 38.7% | 20.8% | 54.2% | 12.3% | 42.8% | 33.8% | 12.9% | 27.9% |
| 17 | 29.6% | 27.7% | 31.0% | 9.3% | 10.0% | 21.8% | 39.9% | 54.6% | 37.4% | 30.5% | 60.0% | 26.0% | 24.7% | 27.6% | 30.2% | 36.5% | 21.0% | 55.6% | 22.1% | 39.2% | 32.9% | 17.8% | 26.7% |
| 18 | 28.7% | 27.3% | 29.6% | 11.3% | 11.2% | 20.6% | 38.5% | 53.0% | 32.6% | 31.3% | 15.4% | 24.3% | 23.1% | 27.1% | 29.7% | 33.1% | 19.3% | 54.7% | 13.8% | 34.5% | 32.6% | 12.7% | 26.3% |
| 19 | 27.5% | 26.1% | 28.5% | 11.4% | 10.0% | 18.9% | 36.0% | 51.9% | 32.3% | 30.2% | 46.2% | 23.2% | 27.0% | 25.8% | 28.7% | 32.6% | 17.7% | 56.0% | 12.4% | 31.1% | 31.3% | 13.5% | 25.1% |
| 20 | 26.4% | 25.2% | 27.2% | 8.1% | 9.8% | 17.7% | 34.4% | 47.2% | 33.2% | 27.9% | 36.4% | 23.4% | 23.5% | 24.2% | 27.8% | 33.8% | 16.8% | 57.5% | 15.8% | 27.3% | 28.1% | 17.0% | 25.9% |
| 21 | 23.9% | 22.1% | 25.3% | 6.7% | 8.8% | 15.4% | 30.5% | 42.8% | 31.4% | 25.7% | 26.3% | 22.4% | 20.8% | 20.9% | 26.3% | 33.7% | 15.1% | 53.9% | 14.5% | 22.7% | 24.9% | 14.7% | 24.9% |
| 22 | 23.4% | 22.3% | 24.3% | 6.3% | 8.7% | 15.3% | 29.5% | 40.7% | 28.4% | 23.9% | 30.4% | 23.6% | 21.0% | 20.2% | 25.5% | 33.9% | 13.9% | 54.1% | 18.6% | 23.5% | 23.5% | 19.1% | 24.2% |
| 23 | 23.4% | 22.1% | 24.5% | 4.8% | 9.3% | 15.5% | 29.7% | 41.3% | 27.9% | 24.2% | 23.6% | 23.3% | 20.3% | 20.3% | 26.7% | 31.1% | 13.1% | 51.3% | 18.1% | 21.3% | 24.7% | 20.5% | 23.9% |
| 24 | 22.1% | 20.5% | 23.4% | 4.7% | 6.9% | 14.6% | 27.9% | 41.0% | 28.1% | 22.5% | 13.2% | 21.4% | 21.9% | 18.9% | 25.3% | 30.3% | 12.2% | 50.0% | 17.9% | 21.1% | 23.6% | 16.8% | 22.7% |
| 25 | 21.2% | 20.3% | 21.9% | 4.5% | 7.8% | 13.5% | 26.9% | 39.9% | 24.9% | 20.6% | 23.1% | 21.8% | 19.1% | 16.7% | 24.8% | 34.4% | 11.6% | 48.0% | 21.5% | 18.7% | 22.1% | 19.0% | 21.5% |
| 26 | 20.7% | 19.6% | 21.5% | 3.3% | 7.1% | 13.3% | 26.5% | 38.5% | 22.7% | 20.1% | 18.3% | 21.9% | 19.3% | 16.1% | 24.8% | 33.6% | 10.9% | 49.0% | 21.2% | 18.7% | 20.8% | 21.9% | 20.6% |
| 27 | 21.2% | 20.2% | 21.9% | 4.5% | 7.2% | 14.1% | 26.7% | 38.8% | 23.5% | 20.3% | 18.2% | 22.7% | 18.3% | 16.4% | 26.2% | 33.2% | 10.5% | 48.8% | 22.0% | 16.5% | 21.2% | 20.8% | 22.0% |
| 28 | 20.8% | 19.9% | 21.5% | 4.3% | 6.1% | 13.4% | 26.6% | 38.0% | 22.3% | 19.0% | 14.5% | 22.7% | 17.7% | 16.2% | 25.1% | 32.9% | 10.5% | 47.9% | 20.4% | 16.9% | 21.0% | 21.8% | 21.2% |
| 29 | 20.1% | 18.7% | 21.2% | 2.8% | 6.4% | 12.7% | 25.6% | 37.1% | 22.6% | 19.0% | 10.3% | 22.0% | 16.5% | 15.5% | 24.4% | 33.9% | 10.3% | 47.2% | 19.8% | 16.9% | 18.8% | 21.7% | 20.9% |
| 30 | 19.9% | 18.6% | 21.0% | 3.1% | 6.5% | 12.4% | 25.1% | 37.1% | 22.8% | 19.0% | 20.0% | 21.3% | 16.7% | 15.5% | 24.0% | 33.2% | 10.3% | 46.4% | 20.2% | 13.2% | 18.7% | 20.9% | 21.4% |
| 31 | 20.5% | 19.3% | 21.5% | 3.9% | 7.8% | 12.5% | 25.4% | 38.4% | 23.4% | 19.6% | 15.7% | 22.0% | 18.2% | 15.9% | 25.4% | 32.7% | 10.6% | 47.2% | 20.1% | 14.2% | 20.2% | 22.2% | 21.1% |
| 32 | 20.5% | 19.5% | 21.2% | 2.9% | 6.2% | 12.9% | 25.8% | 38.1% | 23.9% | 19.3% | 16.4% | 21.4% | 16.0% | 16.0% | 25.1% | 33.0% | 10.8% | 46.5% | 19.7% | 14.7% | 20.1% | 21.4% | 21.5% |
| 33 | 19.3% | 18.6% | 20.0% | 3.2% | 6.1% | 12.3% | 23.7% | 36.4% | 21.9% | 18.8% | 22.6% | 20.7% | 16.6% | 15.0% | 23.0% | 33.0% | 10.3% | 44.3% | 18.4% | 15.4% | 17.6% | 18.9% | 21.4% |
| 34 | 18.9% | 17.7% | 19.8% | 1.8% | 5.5% | 11.8% | 23.3% | 36.7% | 22.4% | 17.3% | 9.8% | 21.2% | 17.4% | 14.3% | 23.5% | 33.2% | 10.3% | 43.5% | 18.0% | 15.1% | 16.7% | 19.9% | 20.5% |
| 35 | 18.5% | 17.6% | 19.2% | 2.5% | 5.7% | 11.0% | 23.6% | 34.5% | 20.9% | 16.4% | 10.3% | 20.2% | 18.3% | 14.5% | 22.5% | 31.4% | 10.3% | 42.5% | 15.6% | 14.0% | 16.5% | 20.2% | 20.4% |
| 36 | 19.0% | 18.2% | 19.7% | 3.3% | 6.9% | 11.3% | 23.4% | 36.0% | 21.9% | 17.1% | 26.7% | 20.6% | 16.4% | 14.6% | 23.2% | 31.2% | 10.7% | 42.6% | 18.0% | 13.9% | 16.9% | 20.3% | 20.7% |
| 37 | 18.9% | 18.5% | 19.3% | 1.9% | 5.8% | 11.8% | 23.7% | 35.6% | 23.6% | 18.5% | 10.0% | 19.9% | 16.6% | 14.8% | 22.6% | 31.8% | 10.6% | 42.5% | 17.0% | 14.1% | 17.2% | 18.5% | 21.0% |
| 38 | 17.8% | 16.7% | 18.9% | 1.6% | 5.3% | 10.6% | 22.2% | 34.9% | 20.9% | 16.6% | 12.9% | 18.9% | 13.9% | 13.2% | 22.9% | 32.2% | 10.1% | 40.9% | 14.3% | 12.1% | 17.5% | 18.3% | 19.6% |
| 39 | 18.2% | 17.9% | 18.5% | 2.1% | 5.8% | 12.1% | 22.7% | 33.1% | 22.9% | 16.7% | 9.5% | 18.9% | 16.4% | 14.3% | 23.0% | 31.6% | 10.2% | 41.6% | 17.4% | 11.3% | 17.1% | 18.7% | 20.0% |
| 40 | 19.5% | 18.5% | 20.3% | 2.1% | 7.2% | 12.4% | 24.1% | 34.6% | 23.9% | 17.9% | 15.4% | 21.0% | 13.8% | 15.6% | 22.3% | 32.9% | 11.1% | 43.8% | 17.0% | 15.3% | 17.4% | 19.4% | 21.6% |
